# The practical effect of “standardized mode” in the construction of community venous catheter maintenance specialty nursing clinics

**DOI:** 10.3389/frhs.2025.1680673

**Published:** 2025-10-14

**Authors:** Zhongling Yang, Fang Fang, Sibei Wan, Ying Gu, Dongting Xu, Yi Sheng, Hong Xing

**Affiliations:** ^1^ Department of Nursing, Shanghai General Hospital, Shanghai Jiao Tong University, Shanghai, China; ^2^ School of Medicine, Tongji University, Shanghai, China

**Keywords:** standardized care model, community health services, PICC maintenance, specialist nursing clinics, clinical outcomes

## Abstract

**Objective:**

To evaluate the impact of establishing standardized community-based venous catheter maintenance nursing clinics, leveraging the training resources and expertise of tertiary general hospitals and following specialized nursing protocols.

**Methods:**

This retrospective before–after study examined the implementation of a standardized peripherally inserted central catheter (PICC) maintenance model across 45 community clinics in Shanghai. The intervention included structured training, supervision, and quality control led by a tertiary hospital vascular access nursing team. Clinical outcomes before (*n* = 831) and after (*n* = 984) the intervention were compared using statistical analysis.

**Results:**

After implementation, the incidence of PICC-related complications decreased significantly (from 7.82% to 0.71%, *P* < 0.001), missed maintenance appointments were reduced (from 2.78 to 0.88, *P* < 0.001), and patient satisfaction improved (from 69.33 ± 7.02 to 88.30 ± 6.92, *P* < 0.001). Moreover, patients experienced decreased travel time, transportation costs, and work absences.

**Conclusion:**

The standardized community-based PICC maintenance model significantly improved care quality, reduced complication rates, and enhanced patient experience. It offers a scalable framework for expanding specialist nursing services within primary care settings.

## Highlights

• A standardized PICC maintenance model was implemented in 45 community clinics across Shanghai.
• PICC-related complication rates significantly decreased post-implementation.
• Patient satisfaction improved notably compared to the pre-implementation phase.
• The model reduced patient economic burden in terms of time, cost, and work absence.
• This community-based approach enhanced the accessibility and continuity of PICC care.

## 1 Introduction

Peripherally inserted central catheters (PICCs) are widely used in patients undergoing chemotherapy, long-term parenteral nutrition, and chronic treatments due to their advantages of prolonged indwelling time, reduced venous injury, and avoidance of repeated venipuncture (1–3). However, studies have shown that if proper care is not maintained after discharge with a PICC, the incidence of catheter-related infections can reach 15%–20%, and the rate of catheter occlusion ranges from 10%–15%, severely compromising treatment continuity and patients’ quality of life (4, 5).

To alleviate the healthcare burden associated with frequent visits to tertiary hospitals, some patients have begun to seek catheter maintenance services at community health service centers (6). However, the current capacity of community-based facilities in PICC care remains limited, with common issues such as non-standardized procedures and insufficient staff training, resulting in suboptimal maintenance quality. Studies have reported that the incidence of PICC occlusion in some community institutions can reach as high as 20%, significantly higher than the rates typically observed in tertiary hospitals, which are often below 5%. This discrepancy may be attributed to inadequate knowledge of PICC management among community healthcare personnel (7, 8). The variation in care quality not only hampers the role of community institutions in providing continued PICC care but also undermines patient trust and acceptance of community-based services. In response to the “hierarchical medical system” policy, several regions have started to explore collaborative PICC care models between tertiary hospitals and community centers. These models aim to enhance primary care capacity, reduce complication rates, and improve patient compliance and healthcare experiences by promoting specialized expertise sharing and the development of standardized care protocols. Although some case reports have described these initiatives, systematic evaluations of such collaborative models are still lacking.

Therefore, this study leveraged the specialized intravenous nursing resources of a tertiary general hospital in Shanghai to develop a collaborative PICC continuous care model between tertiary hospitals and community health service centers. The model was implemented across 45 community nursing clinics citywide. A pre-post comparative study was conducted to systematically evaluate the effectiveness of this model in improving nursing quality, reducing the risk of catheter-related complications, and enhancing patient satisfaction.

## 2 Materials and methods

### 2.1 Study design and setting

We conducted a retrospective before–after evaluation of a standardized, hospital–community collaborative model for PICC maintenance across 45 community nursing clinics in Shanghai, China. The pre-implementation period was January 1, 2016–December 31, 2020; the post-implementation period was March 1, 2021–September 30, 2024, following staged rollout led by a tertiary hospital vascular access nursing team.

#### 2.1.1 Participants and sampling/recruitment

This service evaluation used routinely collected data; no active recruitment or randomization was performed. All patients with a PICC who attended any participating clinic for maintenance during the two windows were included. Baseline demographics and clinical characteristics were summarized to assess comparability.

#### 2.1.2 Data sources and collection procedures

Data were extracted from the community intravenous catheter care information system and standardized nursing records completed at each visit. Variables included de-identified demographics, catheter details, maintenance procedures, and adverse events. Patient-reported outcomes and economic indicators were collected via structured questionnaires under staff guidance.

#### 2.1.3 Data management and quality assurance

A unified documentation template with mandatory fields and logic checks was applied. The tertiary hospital team periodically reviewed records for completeness, performed random checks of complication reporting, and provided feedback, forming a closed-loop management cycle. Data were de-identified prior to analysis; a complete-case approach was adopted for missing key fields.

### 2.2 General information

#### 2.2.1 PICC nursing team and staffing of community clinics

Shanghai General Hospital established China's first PICC nursing clinic in 2002 and formed a specialized intravenous catheter nursing team in 2008 (9). In 2020, the hospital was designated as a national training center for infusion therapy specialist nurses certified by the Chinese Nursing Association. The team currently consists of one chief nurse, four senior nurses, and one staff nurse (10), and in 2020 alone performed approximately 1,800 PICC insertions, demonstrating strong technical capacity and training capability. Under their leadership, 245 nurses were deployed across 45 community nursing clinics in Shanghai between January 2021 and September 2024, including 21 senior nurses, 176 registered nurses, and 48 junior nurses. This combined hospital–community workforce provides the foundation for standardized PICC maintenance through professional training, clinic assessment, and continuous quality oversight delivered via both on-site supervision and remote WeChat-based guidance (11, 12).

#### 2.2.2 Disease characteristics of patients

##### 2.2.2.1 Intervention group

Between March 2021 and September 2024, 984 patients underwent PICC maintenance at the participating community clinics. The group included 436 men and 548 women, with a mean age of 61.3 ± 7.1 years. Gastrointestinal cancers represented the largest diagnostic category with 254 cases, followed by gynecological and breast cancers in 198 patients, lung cancer in 186 patients, and hematologic malignancies in 145 patients. Urological cancers were diagnosed in 86 patients, bone tumors in 48, thyroid cancer in 22, laryngeal cancer in 27, and nervous system tumors in 18.

##### 2.2.2.2 Control group

From January 2016 to December 2020, 831 patients received PICC maintenance in the same clinics. This cohort comprised 375 men and 456 women, with a mean age of 62.4 ± 6.8 years. The diagnostic profile was broadly comparable to that of the intervention group: gastrointestinal cancers were most common with 220 patients, followed by gynecological and breast cancers in 170, lung cancer in 160, and hematologic malignancies in 125. Urological cancers were observed in 75 patients, bone tumors in 40, thyroid cancer in 28, laryngeal cancer in 23, and nervous system tumors in 15.

### 2.3 Establishment of standardized community nursing clinics

#### 2.3.1 Inclusion criteria for community health service centers

Community health service centers participating in the establishment of standardized PICC nursing clinics were required to meet several basic criteria. These included having a dedicated consultation room with a minimum area of 5 m^2^, at least two full-time registered nurses with no less than three years of clinical experience and certified in infusion therapy, availability of a portable ultrasound device, and standardized maintenance kits (13, 14). In addition, centers had to commit to participating in unified training programs and accept ongoing quality control assessments (15).

#### 2.3.2 Quality assessment of community nursing clinics

Before commencing services, each community clinic underwent an on-site environmental and facility assessment conducted by the tertiary hospital's intravenous catheter specialist team to ensure compliance with the construction standards for PICC clinics. During the initial operating phase, senior nurses from the hospital provided direct supervision of the first three maintenance sessions, focusing on admission procedures, aseptic technique, and documentation (16). Any deficiencies identified were addressed immediately through on-site guidance and process optimization. Once clinics demonstrated stable performance, the frequency of quality assessments was gradually reduced from weekly to monthly.

#### 2.3.3 Standardized PICC maintenance training for community nursing clinics

To strengthen the competency and consistency of community-based PICC nurses, all staff were required to complete a standardized training curriculum delivered by the tertiary hospital team before March 2021. The program followed the Training Standards for Infusion Therapy Specialist Nurses issued by the Chinese Nursing Association, supplemented by relevant national guidelines and expert consensus statements. The 12-week course combined online theoretical modules, centralized technical training, supervised clinical practice, and a final stage of independent practice under evaluation. This stepwise approach enabled trainees to transition from theoretical learning to procedural competence, with strict assessments at each stage to ensure readiness for independent clinical practice.

#### 2.3.4 Unified PICC maintenance management system

A unified information management system was established to ensure standardization, systematization, and traceability of PICC maintenance. Each patient's demographic information, catheterization details, and follow-up data were entered promptly into the system. Nursing documentation was completed after every maintenance session using a standardized template, including puncture site condition, catheter patency, and patient-reported symptoms. The system also incorporated a reminder function to improve patient adherence. Data were regularly reviewed by the tertiary hospital team, with feedback provided to community clinics through a closed-loop quality management process.

#### 2.3.5 Implementation procedures and fidelity checks

To guarantee fidelity to the standardized model, community clinics were required to pass environmental and staffing assessments before opening. The first three outpatient sessions were supervised on-site by senior PICC nurses to ensure adherence to admission procedures, aseptic technique, and documentation standards. Continuous oversight was maintained through scheduled audits—initially weekly and later monthly once stable—as well as real-time remote expert support via a WeChat-based collaborative platform. Complex cases, such as suspected catheter-related bloodstream infections or unresolved occlusions, were referred back to the tertiary hospital through a fast-track “green channel.” In addition, monthly patient education sessions were conducted to reinforce self-care and early identification of complications.

### 2.4 Outcome measures

#### 2.4.1 Incidence of PICC-related complications

The primary safety outcome was the occurrence of PICC-related complications during catheter indwelling, including catheter-related bloodstream infections, occlusion, migration, and dislodgement or accidental removal. The incidence was calculated as the proportion of patients experiencing any of these events among the total number observed.

#### 2.4.2 Catheter dwell time

Catheter dwell time was defined as the total number of days from insertion to removal, used to evaluate the stability and durability of catheter function. Mean dwell times were compared between the intervention and control groups.

#### 2.4.3 Unplanned catheter removal rate

Unplanned removal was defined as premature catheter withdrawal due to PICC-related complications such as infection, occlusion, or displacement. The rate was expressed as the proportion of unplanned removals relative to total insertions.

#### 2.4.4 Patient satisfaction

Patient satisfaction was measured with a structured questionnaire developed for nursing service evaluation. The tool contained multiple five-point items covering nursing skills, service attitude, communication efficiency, appointment accessibility, and environmental comfort. Scores were converted to a 0–100 scale, with higher scores indicating greater satisfaction.

#### 2.4.5 Patient adherence

Adherence was defined as completion of PICC maintenance at all prescribed intervals during catheter indwelling. Patients with any missed or delayed visits were categorized as non-adherent.

#### 2.4.6 Economic outcome indicators

Economic outcomes included three patient-reported measures: the total time spent per maintenance visit, the transportation cost per visit, and the number of work absences attributable to PICC maintenance. These data were obtained using a standardized questionnaire during follow-up.

#### 2.4.7 Variable definitions and coding

Complications were analyzed both by individual category and as a composite endpoint, coded as binary outcomes. Adherence was defined as completion of all scheduled maintenance visits on time and coded as adherent, whereas any missed or delayed visits were classified as non-adherent. Patient satisfaction was analyzed as a continuous variable on a 0–100 scale, with higher scores indicating greater satisfaction. Economic indicators were treated as continuous variables, and extreme outliers were identified through distributional diagnostics before analysis.

### 2.5 Statistical analysis

All analyses were performed using SPSS 26.0. Normality was assessed using the Shapiro–Wilk test, and homogeneity of variances was checked when appropriate. Continuous variables were presented as mean ± standard deviation or as median with interquartile range, and group comparisons were conducted using the independent samples t test or the Mann–Whitney U test. Categorical variables were summarized as counts and percentages and compared using the chi-square test or Fisher's exact test. A two-sided alpha level of 0.05 was considered statistically significant. Complete-case analysis was applied for endpoints with missing essential data.

## 3 Results

### 3.1 Comparison of baseline characteristics between the two groups

The baseline characteristics of patients in the two groups are presented in Table 1. There were no statistically significant differences between the groups in terms of age, gender distribution, or cancer types (*P* > 0.05).

**Table 1:** Comparison of baseline clinical characteristics between the two patient groups (*n* = 1,815)

Characteristics		Pre-implementation (*n* = 831)	Post-Implementation(*n* = 984)	*t*/*χ^2^*/*Z*	*P*
Age (years)		61.66(54.19,67.96)	60.79(54.57,67.27)	−0.755	0.450
Gender, *n* (%)	Male	375(45.1)	436(44.3)	0.122	0.727
	Female	456(54.9)	548(55.7)		
Tumor type, *n* (%)	Lung cancer	160(19.3)	186(18.9)	7.215	0.514
	Gastrointestinal cancer	189(22.7)	254(25.8)		
	Hematological malignancy	121(14.6)	145(14.7)		
	Bone tumor	38(4.6)	48(4.9)		
	Urinary system cancer	80(9.6)	86(8.7)		
	Gynecological tumor	165(19.9)	198(20.1)		
	Thyroid cancer	28(3.4)	22(2.2)		
	Laryngeal cancer	35(4.2)	27(2.7)		
	Nervous system tumor	15(1.8)	18(1.8)		
Employment Status, *n* (%)	Employed	403(48.5)	499(50.7)	0.885	0.347
	Retired or Unemployed	428(51.5)	485(49.3)		
Marital status, *n* (%)	Married	540(65.0)	620(63.0)	0.761	0.583
	Unmarried	240(28.9)	300(30.5)		
	Widowed	51(6.1)	64(6.5)		
Education level, *n* (%)	Primary school	170(20.5)	200(20.3)	0.712	0.870
	Junior high school	250(30.1)	280(28.5)		
	Senior high school	210(25.3)	260(26.4)		
	College or above	201(24.2)	244(24.8)		
Income level, *n* (%)	¥2,000–¥5,000	330(39.7)	380(38.6)	0.559	0.756
	¥5,000–¥10,000	290(34.9)	360(36.6)		
	>¥10,000	211(25.4)	244(24.8)		
Medical insurance type, *n* (%)	Self-paid	110(13.2)	140(14.2)	0.853	0.653
	Rural health insurance	380(45.7)	430(43.7)		
	Urban health insurance	341(41.0)	414(42.1)		

### 3.2 Comparison of clinical outcomes, patient satisfaction, and adherence before and after implementation

The overall incidence of PICC-related complications was significantly lower after implementation compared to before (*P* < 0.001). Specifically, the rates of bloodstream infection (*P* < 0.001), catheter occlusion (*P* = 0.005), and catheter dislodgement (*P* = 0.004) showed statistically significant reductions, while the difference in catheter migration was not statistically significant (*P* > 0.05). There was no significant difference in average catheter dwell time between the two groups (*P* > 0.05). The average number of missed or delayed maintenance sessions per patient was significantly lower after implementation (*P* < 0.001). Patient satisfaction scores were significantly higher following implementation compared to pre-implementation (*P* < 0.001). Details are shown in Table 2.

**Table 2:** Comparison of PICC-related outcome indicators before and after implementation of the standardized community-based PICC program (*n* = 1,815)

Indicator		Before implementation (*n* = 831)	After implementation (*n* = 984)	t/*χ*^2^	*P*
PICC complications, *n* (%)	Bloodstream infection	39(4.69)	2(0.20)	41.135	<0.001
	Catheter occlusion	16(1.93)	5(0.51)	7.913	0.005
	Catheter dislodgement	3(0.36)	0(0.00)		0.096
	Catheter accidental removal	7(0.84)	0(0.00)		0.004
	Total	65(7.82)	7(0.71)	59.792	<0.001
Catheter dwell time (days)		128.26 ± 34.57	127.83 ± 34.18	0.262	0.364
Average number of missed scheduled maintenances (times)		2.78(2.41,3.17)	0.88(0.60,1.16)	−36.506	<0.001
Patient satisfaction score		69.33 ± 7.02	88.30 ± 6.92	−57.82	<0.001

### 3.3 Comparison of economic burden indicators before and after implementation

The total round-trip time per maintenance visit was significantly reduced after implementation compared to before (*P* < 0.001). Transportation expenses per visit were also significantly lower following implementation (*P* < 0.001). Additionally, the average number of work absences per person was significantly lower in the post-implementation group (*P* < 0.001). Details are presented in Table 3.

**Table 3:** Comparison of economic outcome indicators before and after implementation of the standardized community-based PICC program (*n* = 1,815)

Economic outcome indicator	Before implementation (*n* = 831)	After implementation (*n* = 984)	Z	*P*
Round-trip time for a single maintenance visit (minutes)	159.61(128.50,190.74)	37.49(32.45,42.79)	−36.305	<0.001
Transportation cost for a single maintenance visit (CNY)	26.61(16.40,36.30)	6.11(3.62,8.49)	−30.576	<0.001
Average total missed workdays per person (times)	5.24(4.38,6.06)	1.75(1.09,2.37)	−35.635	<0.001

## 4 Discussion

### 4.1 Positive impact of standardized clinics on the control of PICC-related complications

The findings of this study demonstrate that the establishment of standardized community PICC maintenance clinics significantly reduced complications, particularly catheter-related bloodstream infections and catheter occlusion, while simultaneously ensuring continuity and safety of care. This indicates that structured nursing models are not only organizational innovations but also have a direct impact on patient safety. Incorporating specialist protocols and continuous quality control into community services provides a practical means of maintaining consistency in vascular access care, which is often lacking in fragmented systems (17).

Kang in a prospective study involving more than fifty thousand catheter days among oncology patients reported that uniform implementation of procedures such as dressing changes, line flushing, and aseptic access was associated with a marked decline in complication rates (4). The present study supports Kang's observations but extends the evidence to the community setting, illustrating that even in environments where training levels are uneven, hospital-level safety can be replicated once standardized procedures are scaled across clinics. Grau compared complication profiles between hospitalized and ambulatory patients and found that outpatients were more prone to occlusion and dislodgement due to inadequate follow-up and inconsistent maintenance (8). Our results are consistent with these findings, as the introduction of structured supervision and reliable follow-up in community clinics substantially reduced these outpatient-specific risks.

Lam studied patients receiving outpatient antimicrobial therapy and identified predictors of catheter occlusion such as delayed flushing and irregular review. These factors were shown to be strong precursors of lumen failure (18). The current study addressed these risks by reducing maintenance delays through geographically accessible community clinics and by introducing systematic reminder mechanisms. In doing so, the standardized model effectively neutralized the risk pathways described by Lam and further illustrated how service reorganization can disrupt the chain of events that lead to failure.

At the same time, this study fills critical gaps left by prior work. Earlier investigations were based on large cohorts but were confined to single hospitals or specific patient populations, or they focused narrowly on antimicrobial therapy. None of these studies assessed a city-wide intervention that combined staff training, patient education, routine audit, and remote supervision. By documenting reductions in complications across forty-five clinics under everyday practice conditions, the present study demonstrates that these benefits are not confined to specialized environments but can also be reproduced in decentralized, routine community care.

### 4.2 Impact of standardized clinics on patient adherence and satisfaction

This study confirms that a standardized community PICC model improves adherence and satisfaction under real-world conditions. Missed and delayed maintenance visits decreased and patient ratings increased, which indicates closer alignment between service cadence and daily life and a stronger perception of reliability at the point of care. These outcomes are not ancillary to safety. They are mechanisms that sustain timely maintenance, early signal reporting, and effective self-care and they therefore support the reduction in complications. International guidance on outpatient intravenous therapy likewise emphasizes systematic monitoring, clear patient education, and programmatic oversight to maintain engagement and on-schedule follow-up (19, 20).

This study advances evidence from oncology settings into system-level implementation. Zhu reported that continuous nursing interventions improved self-management, extended catheter dwell time, reduced complications, and increased both adherence and satisfaction (21). We observed similar gains across a citywide community network, which indicates that when unified procedures and continuous supervision are implemented at scale, technical standards can be converted into everyday practice and the gap between written standards and real-world execution can be closed.

Improvements in adherence were achieved through three pathways. First, proximity and rapid response reduced the time and travel burden for each visit, lowered the personal cost of keeping appointments, and increased the probability of on-time attendance. Second, unified procedures delivered by credentialed staff produced a stable care experience and strengthened trust. Third, individualized education and remote support embedded in the community platform supplied knowledge and confidence for self-care between visits. Evidence from digital education programs shows that structured multimedia reinforcement improves knowledge, raises satisfaction, and reduces delays in seeking care after early complications, which is consistent with the support pathway used in this study (22, 23).

Improvements in satisfaction emerged across complementary dimensions. Goes indicated that patient satisfaction is highly sensitive to the quality of nursing processes rather than outcomes alone (24). In our setting, greater accessibility, more frequent contact between nurses and patients, and clearer communication likely raised perceived process quality. Workforce capacity is a critical lever. Zhang reported that building multiskilled community nursing teams and formalizing community clinics in Shanghai aligned services with patient expectations and improved experience (25). Qualitative work from the United Kingdom documented high ratings for outpatient antimicrobial services and identified communication and coordination as priorities for further improvement, which mirrors the emphasis in our model on standardized handoffs and continuous feedback (26).

Broader evidence links adherence and satisfaction to specific design features of care. Hamad reported that adherence is shaped by communication with clinicians, regimen complexity, and social support. Reliable follow-up, patient-friendly scheduling, and accessible advice channels can target these determinants and these elements are built into the current model (27). Guidance also emphasizes that outpatient services require complete infrastructure for monitoring, escalation, and education rather than *ad hoc* arrangements. The present study shows that when these components operate together at community level, adherence and satisfaction rise in parallel with safety (28).

### 4.3 Impact of standardized clinics on patients’ economic burden

PICC is essential for patients with cancer and chronic disease, yet long term maintenance often leads to cumulative hidden costs. Under traditional models patients travel frequently to tertiary hospitals for routine maintenance, which generates repeated transport expenses, time loss, and work absences. In our evaluation, after the standardized community clinic model was implemented, time per maintenance visit, transportation costs, and average work absences per person decreased significantly, with statistical significance at P less than 0.001. These changes indicate tangible relief of financial pressure at the household level and closer alignment of service cadence with daily life.

This direction is consistent with research on access and affordability in primary care. Li reported that bringing services closer to residents is associated with higher satisfaction and lower indirect expenses such as reduced travel and waiting, which supports shifting maintenance tasks to community clinics (29). Bedford showed in a United Kingdom nursing journal that placing PICC services in community settings under nurse leadership reduces time burden and improves access, which is a practical lever to strengthen adherence and control costs (30). Value does not come from access alone. Nurse led structured care can create gains through education, engagement, and protocol driven follow up. Doherty demonstrated in a randomized trial that nurse led programs that emphasize patient engagement and goal directed strategy deliver superior clinical value and cost effectiveness (31). The same principle applies to catheter maintenance, which is characterized by low complexity and repeated contacts (32).

Economic gains likely arise through two pathways. The first pathway is a reduction in direct costs that comes from shorter travel and visit time and fewer missed workdays. The second pathway is indirect savings created by a decline in adverse events. Irregular maintenance and interruptions in care are widely recognized risk factors for catheter related infection and occlusion, which trigger subsequent visits, device replacement, and antimicrobial therapy (33). In this program, the concurrent improvement in complications and adherence suggests a double dividend in which both direct spending and complication related expenditure decline for patients and for the health system. These results have system level implications. Once community clinics meet readiness standards and operate unified workflows, capacity can be localized without sacrificing technical quality, which is essential for equitable access and for the sustainability of oncology and chronic care. The model also aligns with hierarchical care by reserving hospital resources for complex needs and keeping routine maintenance in the community.

## 5 Limitations and future directions

This study has some limitations. The retrospective before–after design restricts causal inference, and reliance on routine records and patient questionnaires may introduce bias. Generalizability is limited to regions with similar workforce capacity and clinic readiness.

Future research should adopt prospective multicenter designs to strengthen causal inference and external validity. Economic evaluations should include detailed micro-costing, and digital tools such as remote monitoring deserve further testing. Patient-centered outcomes should also be broadened to capture treatment burden and financial impact.

## Data Availability

The original contributions presented in the study are included in the article/Supplementary Material, further inquiries can be directed to the corresponding author.
